# Documentation Quality and Educational Value in AI-Assisted Feedback

**DOI:** 10.2196/105029

**Published:** 2026-08-26

**Authors:** Amane Endo, Takeshi Kimura, Yuki Kataoka

**Affiliations:** 1 Department of Medical Education, Faculty of Medicine, Juntendo University, 2-1-1 Hongo, Bunkyo-ku, Tokyo, Japan, 81 3-3813-3111; 2 Center for Medical Education, Graduate School of Medicine, Nagoya University, Nagoya, Japan; 3 Center for Postgraduate Clinical Training and Career Development, Nagoya University, Nagoya, Japan; 4 Scientific Research WorkS Peer Support Group (SRWS-PSG), Osaka, Japan

**Keywords:** ambient scribe, artificial intelligence, feedback, medical student education, formative assessment, competency-based education, AI

Talwalkar and colleagues [1] address an important problem in medical education: faculty often observe learner performance but struggle to translate those observations into written feedback. Their randomized evaluation of an ambient AI scribe workflow is timely and practically relevant. The study is also valuable because it examines a real educational workflow, includes instructor review of AI-generated notes, and explicitly evaluates unedited AI summaries for mischaracterization and hallucination. These features make the study an important contribution to the emerging literature on AI-assisted feedback documentation. However, there are concerns regarding the interpretation of the results.

The central concern is that a well-documented AI-assisted feedback note should be distinguished from feedback that is educationally effective for learners. Their findings show that AI assistance improved the quality of written feedback documentation, as measured by the Evaluation of Feedback Captured Tool (EFeCT) score [1]. The EFeCT assesses whether specific feedback elements are present in the written note. It does not assess whether students understood, accepted, trusted, or used the feedback. This distinction matters because feedback becomes educationally meaningful only when learners can engage with it and use it to guide future action. Students’ use of feedback depends on self-regulation, beliefs, emotions, and their ability to identify next steps [2]. Molloy and colleagues [3] similarly caution against treating feedback as a simple input separated from learner involvement and effects beyond the immediate task. Thus, higher EFeCT scores should be understood as evidence of improved documentation quality unless learner uptake and subsequent performance are also examined.

A second concern is that narrative length may have influenced the score difference. As Talwalkar and colleagues [1] also note, human-only narratives were much shorter than AI-assisted outputs. Because the EFeCT awards one point for each feedback element present, longer notes have more opportunity to contain those scored elements and may, therefore, receive higher scores even when they are no more useful to learners. This matters for implementation. If institutions adopt AI tools because they improve documentation metrics, they may produce feedback records that receive higher scores while being more cognitively demanding and harder for learners to process. Shute’s [4] review supports feedback that is specific and clear but also emphasizes that elaborated feedback should remain manageable for learners.

Related evidence supports this interpretation. Kondo and colleagues [5] found that AI-generated feedback on clerkship logs was longer and more consistent, whereas supervisor feedback drew on clinical context and professional judgment. This suggests that AI may improve structure and consistency, while human supervisors may provide contextual educational judgment. These are not interchangeable qualities, but they may be complementary.

Taken together, these considerations highlight the need to distinguish documentation quality from educational usefulness in future research. In addition to documentation metrics, evaluations should include learner understanding, perceived actionability, trust, feedback use, subsequent performance, and the burden associated with reading longer feedback. These outcomes would clarify whether AI-assisted feedback truly improves learning.

## Abbreviations

EFeCT: Evaluation of Feedback Captured Tool
